# Activity and expression of urokinase-type plasminogen activator and matrix metalloproteinases in human colorectal cancer

**DOI:** 10.1186/1471-2407-6-211

**Published:** 2006-08-18

**Authors:** Tae-Dong Kim, Kyoung-Sub Song, Ge Li, Hoon Choi, Hae-Duck Park, Kyu Lim, Byung-Doo Hwang, Wan-Hee Yoon

**Affiliations:** 1 Department of Surgery, College of Medicine, Chungnam National University and Hospital, Daejeon, 301-721, Korea; 2 Department of Biochemistry, College of Medicine, Chungnam National University, Daejeon, 301-721, Korea; 3 Cancer Research Institute, Chungnam National University, Daejeon, Korea

## Abstract

**Background:**

Matrix metalloproteinase-2 (MMP-2), matrix metalloproteinase-9 (MMP-9), and urokinase-type plasminogen activator (uPA) are involved in colorectal cancer invasion and metastasis. There is still debate whether the activity of MMP-2 and MMP-9 differs between tumors located in the colon and rectum. We designed this study to determine any differences in the expression of MMP-2, MMP-9 and uPA system between colon and rectal cancer tissues.

**Methods:**

Cancer tissue samples were obtained from colon carcinoma (n = 12) and rectal carcinomas (n = 10). MMP-2 and MMP-9 levels were examined using gelatin zymography and Western blotting; their endogenous inhibitors, tissue inhibitor of metalloproteinase-2 (TIMP-2) and tissue inhibitor of metalloproteinase-1 (TIMP-1), were assessed by Western blotting. uPA, uPAR and PAI-1 were examined using enzyme-linked immunosorbent assay (ELISA). The activity of uPA was assessed by casein-plasminogen zymography.

**Results:**

In both colon and rectal tumors, MMP-2, MMP-9 and TIMP-1 protein levels were higher than in corresponding paired normal mucosa, while TIMP-2 level in tumors was significantly lower than in normal mucosa. The enzyme activities or protein levels of MMP-2, MMP-9 and their endogenous inhibitors did not reach a statistically significant difference between colon and rectal cancer compared with their normal mucosa. In rectal tumors, there was an increased activity of uPA compared with the activity in colon tumors (P = 0.0266), however urokinase-type plasminogen activator receptor (uPAR) and plasminogen activator inhibitor-1 (PAI-1) showed no significant difference between colon and rectal cancer tissues.

**Conclusion:**

These findings suggest that uPA may be expressed differentially in colon and rectal cancers, however, the activities or protein levels of MMP-2, MMP-9, TIMP-1, TIMP-2, PAI-1 and uPAR are not affected by tumor location in the colon or the rectum.

## Background

Colorectal cancer, one of the most prevalent cancers worldwide [1], is the second leading cause of cancer-related mortality in developed countries [2]. Tumor cell invasion and metastasis are regarded as multi-step phenomena, involving the proteolytic degradation of the basement membrane (BM) and the extracellular matrix (ECM), altered cell adhesion, and the physical movement of tumor cells. Among the many steps in invasion and metastasis, excessive degradation of the matrix is one of the hallmarks of this process [3].

Many proteinases are capable of degrading ECM components, but the proteinase system primarily responsible for ECM degradation in vivo are matrix metalloproteinase (MMPs) and plasminogen activator (PA) systems [3,4]. These proteinases have been closely linked with the invasive and metastatic phenotype of cancer cells [5,6].

MMP-2 and MMP-9 have been implicated to play a role in colorectal cancer progression, invasion and metastasis in animal models and patients [7]. The enzyme activity is regulated extracellularly and its regulation is mainly based on the balance between pro-enzyme activation and inhibition by tissue inhibitors of MMPs (TIMPs) [8].

Urokinase plasminogen activator (uPA) is a 55 kDa serine protease, which is secreted as an inactive pro-enzyme (pro-uPA). It seems that activation of pro-uPA mostly occurs after binding to its receptor uPAR (uPA receptor). Plasminogen activator inhibitors (PAI-1 and PAI-2) inhibit both receptor-bound and free uPA [9]. uPA is found in cellular structures at the leading edge of migrating cells that are involved in adhesion, migration, invasion, and intravasation [10]. The uPA system is considered to be a marker for malignancy in several types of cancer including colorectal cancer [11-13].

Observations support the theory that development of colon and rectal cancers may involve different mechanisms. Konishi et al. [14] reported that there may be some mechanisms promote the progression of mucosal lesions to invasive cancers in the left colon and rectum, whereas a de novo pathway from depressed type lesions may be implicated in some cancers of the right colon. Kapiteijn et al. [15] found that rectal cancers showed significantly more nuclear β-catenin and p53 expressions than colon cancers. In addition, there was a report which shown notable overexpression of COX-2 protein in tumors located in the rectum compared with other locations in the colon [16].

In terms of MMPs expressions in colorectal cancers, Liabakk et al. [17] reported no significant difference in MMP-2 and MMP-9 levels between tumors located in the colon or the rectum. However, Roeb et al. [18] demonstrated differences in MMP-9 activity between colon and rectal cancers. These studies have looked at only the MMP system. No previous study has determined the expression of these two proteinase systems in the different sites of colon and rectal cancer.

Therefore, we classified colorectal cancer into colon and rectal cancer and examined differences in the expression of MMP-2, MMP-9, TIMP-1, TIMP-2, uPA, uPAR and PAI-1 between colon and rectal cancer.

## Methods

### Tissue samples

This study was approved by the Institutional Review Boards of the Chungnam National University Hospital. Tissue samples were obtained from sequential patients during elective operations for colon (n = 12) and rectal (n = 10) cancers. The resected specimens were washed with cold saline. After removal of mucus with sterile gauze, tumor tissue was excised and corresponding normal colonic epithelial cell layer located at least 5 cm from the tumor was scraped with sterile slide glass and collected. All tissues were immediately frozen in liquid nitrogen and then stored at -70°C until analysis.

### Tissue homogenates

Frozen tumor tissue and normal mucosa were thawed and homogenized in 100 mM Tris-HCl (pH 8.0) and 1% Triton X-100. Tissue homogenates were centrifuged at 1,500 g for 15 minutes to remove debris. Following clarification, the homogenates were centrifuged at 100,000 g for 1 h at 4°C. Protein concentration of homogenates was determined for each sample using the Bradford method (Bio-Rad, Hercules, CA) [19].

### Analysis of MMP-2 and MMP-9 activities by gelatin zymography

Gelatinase activities of MMP-2 and MMP-9 in tissue homogenates were examined by gelatin zymography. The tissue homogenates (2 μg protein/lane) were separated by electrophoresis in 10% polyacrylamide gel impregnated with 1 mg/ml of gelatin (Fisher Chemical Co., Fair Lawn, NJ) under nonreducing conditions. After electrophoresis, the gels were washed twice in 2.5% Triton-X 100 for 30 min, incubated with reaction buffer (50 mM Tris-HCl, 5 mM CaCl_2_, and 0.02% NaN_3_, pH 8.0) for 18 h at 37°C, and stained with Coomassie blue G-250. Conditioned media of HT-1080, which have the highest baseline MMP-9 and MMP-2 activities, were used as a positive control in gelatin zymography. The Bio-Rad Gel-Doc system (Gel Doc 2000™ Documentation System and QuantityOne-software, Bio-Rad, Hercules, CA) was utilized to quantify zymography band intensities. The relative activity of MMP-2 and MMP-9 was expressed as the sum of the pro-enzyme and the active form, since active form was faintly present in normal colon and rectal mucosa.

### Analysis of uPA activities by casein-plasminogen zymography

Activities of uPA in tissue homogenates were examined by casein-plasminogen zymography as previously described [20]. The tissue homogenates (10 μg protein/lane) were separated by electrophoresis in 10% polyacrylamide gel copolymerized with 1 mg/ml of casein (Sigma, St. Louis, MO) and 13 μg/ml human plasminogen (Sigma, St. Louis, MO) under nonreducing conditions. After electrophoresis, the gels were washed twice in 2.5% Triton-X 100 for 30 min, incubated with reaction buffer (50 mM Tris-HCl, 5 mM CaCl_2_, and 0.02% NaN_3_, pH 8.0) for 18 h at 37°C, and stained with Coomassie blue G-250. Conditioned media of HT-1080, which have the highest baseline uPA activities, were used as a positive control in casein-plasminogen zymography.

### Western blot analysis

Equal amounts of protein (100 μg) were loaded in each lane and separated by 10–15% SDS-PAGE. The proteins were transferred to PVDF membranes (Millipore, Billerica, MA). The membranes were blocked with 5% skim milk in TBS containing 0.1% Tween 20, and then incubated at room temperature for 3 h in the presence of each antibody (mouse monoclonal anti-human MMP-2 obtained from Oncogene, Boston, MA, mouse monoclonal anti-human MMP-9, anti-human TIMP-1 and anti-human TIMP-2 obtained from Neomarkers, Fremont, CA). The membrane was washed three times with 0.1% Tween 20 in PBS and stained with horseradish peroxidase-conjugated secondary antibodies. The ECL system (Amersham Life Science, Buckinghamshire, UK) was used for detection [21]. The intensities of immunoreactive bands were measured by computerized image analysis (QuantityOne-software, Bio-Rad, Hercules, CA) and normalized to actin as the internal control.

### uPA, uPAR andPAI-1 concentrations by ELISA assay

Biochemical analyses of uPA, uPAR, and PAI-1 were performed using the sandwich ELISA which employs murine monoclonal anti-human uPA, rabbit polyclonal anti-human uPAR or anti-human PAI-1 antibodies coated on 96-well plates (IMUBIND, American Diagnostica Inc., Greenwich, CT). The uPA assay recognizes pro-uPA, high molecular-weight uPA, receptor-bound uPA and uPA complexed with PAI-1 and PAI-2. With the uPAR assay, both soluble and native (membrane-associated) uPAR as well as complexes of either uPAR/uPA or uPAR/uPA/PAI-1 are all recognized. The PAI-1 assay determines both active and inactive forms of free PAI-1 and PAI-1 complexes. Extracts were diluted 1:20 in assay buffer, and 100 μl aliquots of each extract were incubated overnight at 4°C in precoated microtest wells. Wells were washed thoroughly using wash buffer, and a secondary biotinylated antibody that recognizes a specific epitope on uPA, uPAR, or PAI-1 molecules was added for each analysis. Wells were washed again after an incubation of 1 h, and 100 μl of enzyme conjugate was added (streptavidin-conjugated horseradish peroxidase), leading to the formation of the antibody-enzyme detection complex. After a 1 h incubation, wells were washed again. Then 100 μl of perborate 3, 3', 5, 5'-tetramethylbenzidine substrate was added to each well and reacted with horseradish peroxidase, producing a blue solution. Sulfuric acid (0.5 M) was used as a stopping solution, which yielded a yellow color in the reaction. The levels of uPA, uPAR, and PAI-1 were quantified by measuring the absorbance at 450 nm, using a microplate reader and interpolating them with absorbance of the standard curve. The final concentration was expressed in ng of uPA, uPAR, and PAI-1 per mg extract protein.

### Statistical analysis

Statistical analyses were performed using the The Graphpad Instat version 3.05 software program (Graphpad Software, Inc, San Diego, CA). Significance of difference between values was assessed using paired t-test and all values were expressed as mean ± SE. Statistical significance was assigned when P < 0.05.

## Results and discussion

### Patients characteristics

The patient's age, sex, tumor stage, differentiation and lymphovascular invasion status were summarized in Table 1.

### Activities of MMP-2 and MMP-9

**Figure 1:**
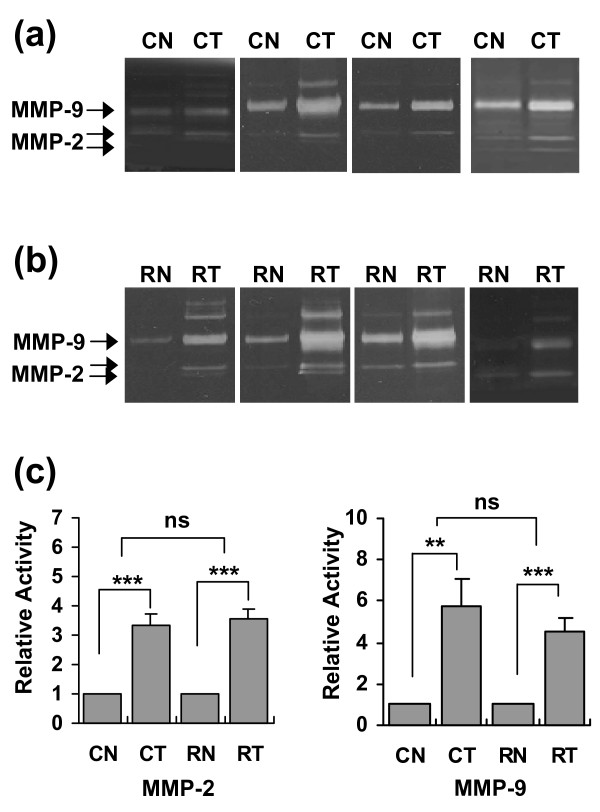
**MMP-2 and MMP-9 activities analyzed by gelatinzymography**. (a) Gelatin zymography of 4 representative specimens of colon tumor (CT) and the corresponding paired adjacent normal colonic mucosa (CN) from colon cancer patients. (b) Gelatin zymography of 4 representative specimens of rectal tumor (RT) and the corresponding paired adjacent normal rectal mucosa (RN) from rectal cancer patients. (c) Zymographic band densities from all tissue samples were quantified by densitometry. The relative enzyme activities of MMP-2 and MMP-9 are expressed as a mean ± SE. ns, non-significant. **P < 0.01. ***P < 0.001

Zymographic detection on substrate gels is often a good choice for the initial characterization of a proteinase activity. This technique permits important information, such as estimated molecular weight and proteinase class, to be obtained. In addition, it circumvents two common problems encountered in the assay: (a) Many proenzymes show activity when assayed in this manner, thus eliminating the requirement for activation. (b) Electrophoresis can result in the separation of non-covalent proteinase-inhibitor complexes. We performed gelatin zymography for the initial characterization of MMP-2 and MMP-9 in tumors and their corresponding normal mucosa from colon and rectal cancer patients (Figure 1). After zymography, four gelatinolytic bands which inhibited with MMPs inhibitor, 1,10-phenanthroline could be observed, with molecular masses of 92 kDa (latent MMP-9), 84 kDa (active MMP-9), 72 kDa (latent MMP-2), and 62 kDa (active MMP-2). (Figure 1a,b). Computer assisted image analysis of the intensity of the proteolytic bands showed that the levels of MMP-2 and MMP-9 activity in colorectal cancer tissues were higher than those in normal tissues in all samples (22/22). The increase in MMP-2 and MMP-9 activity was 3.3-fold (P = 0.0002) and 5.8-fold (P = 0.0042) in colon cancer tissue compared with normal colonic mucosa, respectively (Figure 1c). In rectal cancer tissue, the levels of MMP-2 and MMP-9 activity were 3.5-fold (P < 0.0001) and 4.5-fold (P = 0.0006) higher than in normal mucosa, respectively (Figure 1c). Roeb et al. [18] reported that in colon tumors, MMP-9 activity was increased significantly compared with normal tissue, but in rectal tumors there were no significant differences in MMP-9 levels between tumors and normal tissues, showing a difference between tumors located in colon and rectum. However, in our zymography results, the levels of MMP-2 and MMP-9 activity were increased in the rectal cancer tissues as well as in the colon cancer tissues. Furthermore, no significant difference of the increase of either MMP-2 or MMP-9 activity was found between colon and rectal cancer tissues (MMP-2, P = 0.9815; MMP-9, P = 0.7979; Figure 1c).

### Protein expression of MMP-2 and MMP-9

**Figure 2:**
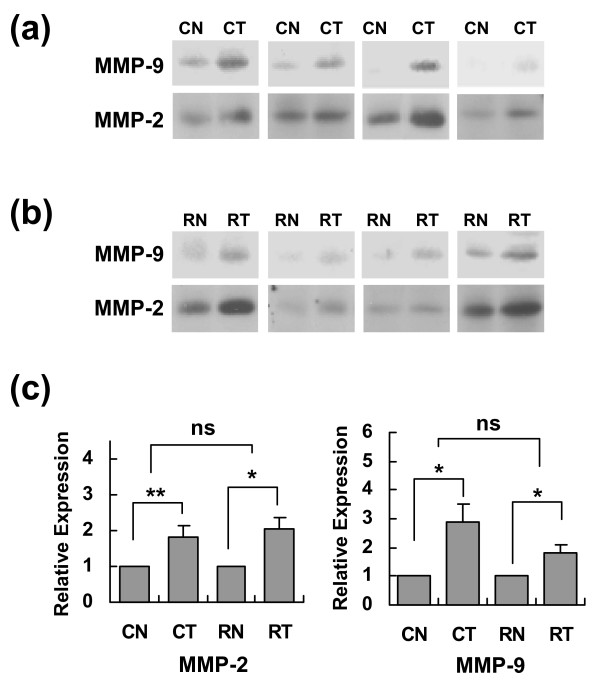
**The expression of MMP-2 and MMP-9 in tumor tissuesand adjacent normal mucosa of colon and rectal cancer patients**. (a) Representative Western blot analysis of colon tumor (CT) and the corresponding paired adjacent normal colonic mucosa (CN) from colon cancer patients. (b) Representative Western blots of rectal tumor (RT) and the corresponding paired adjacent normal rectal mucosa (RN) from rectal cancer patients. (c) Band densities from all tissue samples were quantified and the relative expression of MMP-2 and MMP-9 are expressed as a mean ± SE. ns, non-significant. *P < 0.05. **P < 0.01.

To further investigate the difference in protein levels of MMP-2 and MMP-9, Western blotting was used. A single band was found at 72 kDa for MMP-2 and at 92 kDa for MMP-9 in each sample (Figure 2a,b). Image analysis of the intensity of the bands showed that the increase in MMP-2 and MMP-9 was 1.8-fold (P = 0.0041) and 2.9-fold (P = 0.0175), respectively, in colon cancer tissue compared with normal colonic mucosa (Figure 2c). In rectal cancer tissue, the levels of MMP-2 and MMP-9 were 2.0-fold (P = 0.0477) and 1.8-fold (P = 0.0219) higher than in normal mucosa, respectively (Figure 2c). Furthermore, consistent with results obtained by previous zymographic analyses (Figure 1), no significant difference in the increase of MMP-2 protein expression was found between colon cancer and rectal cancer tissue (P = 0.7383; Figure 2c, left panel). There was a trend toward higher MMP-9 expression in colon tumors than in rectal tumors when compared to the corresponding paired adjacent normal tissues, but the difference was not statistically significant (P = 0.1747, Figure 2c, right panel). These results suggest that MMP-2 and MMP-9 are expressed increasingly in equal amount and activity regardless of tumor location in colon and rectum compared with corresponding normal mucosa.

### Protein expression of TIMP-1 and TIMP-2

**Figure 3:**
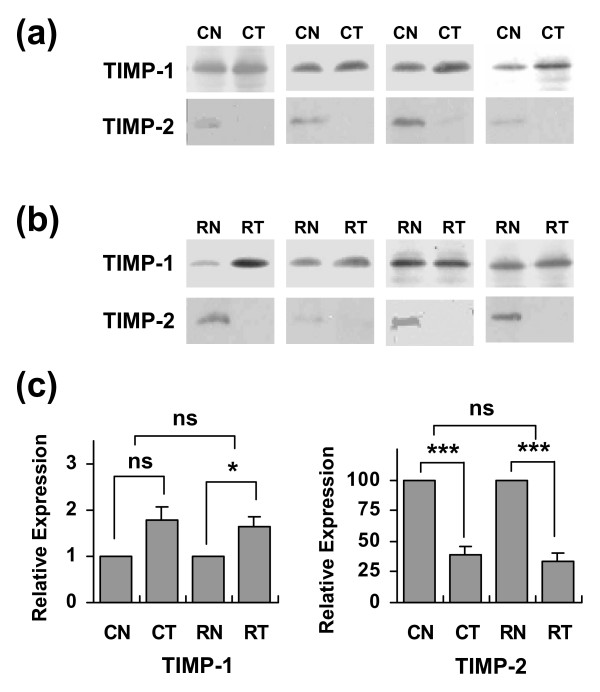
**The expression of TIMP-1 and TIMP-2 in tumortissues and adjacent normal mucosa of colon and rectal cancer patients**. (a) Representative Western blot analysis of colon tumor (CT) and the corresponding paired adjacent normal colonic mucosa (CN) from colon cancer patients. (b) Representative Western blots of rectal tumor (RT) and the corresponding paired adjacent normal rectal mucosa (RN) from rectal cancer patients. (c) Band densities from all tissue samples were quantified, and the relative expression of TIMP-1 and TIMP-2 are expressed as a mean ± SE. ns, non-significant. *P < 0.05. ***P < 0.001.

MMP enzyme activity is regulated at multiple levels, including activation of the latent precursor, gene transcription, and specific inhibition by naturally occurring tissue inhibitors [8]. To determine the expression of the endogenous inhibitors of MMP-2 and MMP-9 in colon and rectal cancer tissues, we examined the protein expression of TIMP-2 and TIMP-1 by Western blot (Figure 3). TIMP-1 immunoreactive band (30 kDa) was identified in all normal mucosa samples and its intensity seemed to be increased in both cancer tissues, on the other hand, TIMP-2 immunoreactivity (21 kDa) was detected in 100% of normal mucosa regardless of tumor location and was hardly detected or absent in colon (83.3%) and rectal (100%) cancer tissues. (Representative data shown in Figure 3a,b). Image analysis showed that TIMP-1 levels seemed to be higher in the colon cancer tissues (1.82 ± 0.32-fold, P = 0.0729) and were significantly higher in the rectal cancer tissues (1.64 ± 0.23-fold, P = 0.0477) than in the corresponding normal mucosa (Figure 3c, left panel). Upregulated TIMP-1 expression in cancer tissues, including colorectal cancer, was observed in other reports [22,23]. Thus, upregulation of TIMP-1 in cancer tissue may be a common feature in human cancer. In contrast, TIMP-2 expression in colon and rectal cancer tissues was significantly lower than in corresponding normal mucosa (38.84%, P = 0.0008; 33.82%, P = 0.0004, respectively) (Figure 3c, right panel). It was reported that TIMP-2 was regulated independently of TIMP-1, both in cell culture and in vivo [24]. Previously published studies reported that the expression level of TIMP-2 in colorectal carcinoma tissues was much lower than that in normal tissues [23,25]. Our data confirm the down-regulation of TIMP-2 in colorectal cancer as compared to normal tissue. Moreover, we found no significant difference between colon and rectal cancer tissues in terms of the expression of TIMP-1 (P = 0.7873) and TIMP-2 (P = 0.6802) (Fig. 3c).

### Activity and protein expression of uPA

**Figure 4:**
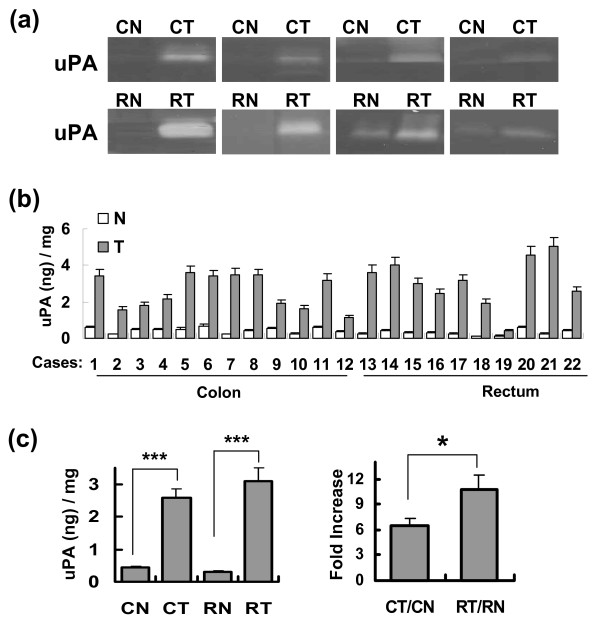
**Activity and expression of uPA in tumor tissues andadjacent normal mucosaof colon and rectal cancer patients**. (a) Casein-plasminogen zymography of representative specimens of colon tumor (CT) and the corresponding paired adjacent normal colonic mucosa (CN) from colon cancer patients, and rectal tumor (RT) and the corresponding paired adjacent normal rectal mucosa (RN) from rectal cancer patients. (b) Concentration of uPA in tumor tissues and adjacent normal colonic mucosa of colon and rectal cancer patients determined by ELISA. (c) Mean concentration (left panel) and fold increase (right panel) of uPA in tumor tissues compared with normal tissues. uPA levels are significantly increased in rectal cancer patients compared with colon cancer patients. *P < 0.05. ***P < 0.001.

Casein-substrate gels copolymerized with plasminogen were used to characterize uPA activity present in tumor tissues and corresponding normal tissues. As shown in Figure 4a, a lysis band corresponding to a molecular weight of approximately 55 kDa was identified as uPA in tumor lysates; however, very little or no activity was detected in normal mucosa. Furthermore, the increase in the activity of uPA in colon cancer tissues compared with normal mucosa was lower than that of rectal cancer.

To further evaluate uPA expression, ELISA was performed. uPA levels in colon cancer and rectal cancer tissues (median value: 2.57 ± 0.27 ng/mg protein, 3.08 ± 0.42 ng/mg protein; range: 1.15–3.63 ng/mg protein, 0.41–5.02 ng/mg protein, respectively) were significantly higher than that of corresponding normal mucosa (median value: 0.44 ± 0.043 ng/mg protein, 0.31 ± 0.044 ng/mg protein; range: 0.21–0.66 ng/mg protein, 0.11–0.58 ng/mg protein, respectively) (P < 0.001) and found to be elevated in 100% (22/22) of colorectal cancer tissues, when compared with corresponding normal mucosa (Figure 4b). The levels of uPA were increased in colon tumor tissues (6.42-fold), as well as in rectal tumor tissues (10.78-fold), compared with corresponding normal mucosa (Figure 4c). Furthermore, there was a statistically significant difference in the increasing rate of uPA protein between colon and rectal cancers (P = 0.0266) (Figure 4c, right panel).

Upregulated uPA levels appear to be an important negative prognostic indicator in colorectal cancer [9]. A previous study suggested that the stromal cells were responsible for the increased uPA [26]. However, other studies demonstrated production of uPA only by the cancerous epithelial cells [27,28]. It appears that there are multiple pathways present in the cancerous epithelial cell that lead to overproduction of uPA [29]. It has been suggested that the NF-kB activation pathway [30] and cyclooxygenase-2 (COX-2) [31] lead to increased uPA production. In addition, Dimberg et al. [16] reported that the enhanced COX-2 protein levels were localized more preferentially to rectal tumors than colon tumors. In the present study, we found that there was significant difference in uPA protein levels between colon and rectal cancer tissues. The explanation and interpretation of this finding is still uncertain but may be due to differences in uPA regulatory genes and/or differences in molecules such as COX-2 in the colon and rectal cancer tissues.

### Protein expression of uPAR and PAI-1

**Figure 5:**
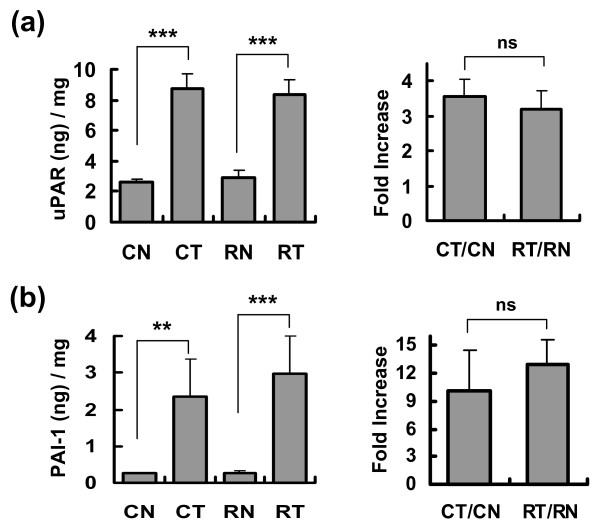
**Expression of uPAR and PAI-1 in tumor tissues andadjacent normal mucosa of colon and rectal cancer patients**. Mean concentration of uPAR (a, left panel) and PAI-1 (b, left panel) determined by ELISA. Fold increase of uPAR (a, right panel) and PAI-1 (b, right panel) in tumor tissues compared with normal tissues, showing no significant difference of increase in colon tumor and rectal tumor. ns, non-significant. **P < 0.01. ***P < 0.001.

ELISA analysis was used to investigate the protein expression of uPAR and PAI-1 (Figure 5). uPAR and PAI-1 levels of colon cancer (median value: 8.72 ± 0.97 ng/mg protein, 2.35 ± 1.011 ng/mg protein; range: 5.10–13.38 ng/mg protein, 0.42–12.76 ng/mg protein, respectively) and rectal cancer tissues (median value: 8.38 ± 0.94 ng/mg protein, 2.99 ± 0.99 ng/mg protein; range: 4.06–14.33 ng/mg protein, 0.495–11.31 ng/mg protein, respectively) were significantly higher (P < 0.01) than that of normal colonic mucosa (median value: 2.61 ± 0.23 ng/mg protein, 0.25 ± 0.029 ng/mg protein, respectively; range: 1.43–3.78 ng/mg protein, 0.07–0.38 ng/mg protein, respectively) and normal rectal mucosa (median value: 2.96 ± 0.41 ng/mg protein, 0.26 ± 0.053 ng/mg protein, respectively; range: 1.85–5.36 ng/mg protein, 0.05–0.57 ng/mg protein, respectively) and these proteins were found to be elevated in 100% (22/22) of colon and rectal cancer tissues, when compared with corresponding paired normal mucosa (Figure 5a,b, left panels). The levels of uPAR and PAI-1 were increased in colon cancer (3.56-fold, 10.08-fold, respectively) and rectal cancer (3.19-fold, 12.90-fold, respectively), compared with normal mucosa (Figure 5a,b, right panels). However, the increase in uPAR and PAI-1 levels did not reach a statistically significant difference between colon and rectal cancer (uPAR, P = 0.6207; PAI-1, P = 0.5781).

The functional role of PAI-1 in cancer tissue is unknown. Recently, it was reported that the increase of PAI-1 expression was observed in node-positive and metastatic cancers compared with node-negative and metastasis-negative cancers [32]. The upregulated PAI-1 in malignant tumor may play a role in protecting the tumor tissue against the destruction of uPA, in reimplantation of circulating tumor cells, and in promoting angiogenesis [12].

The number of patients in this study was quite small to reveal the significance of statistical analysis between each tumor status and various enzymes with related proteins we have studied. No association among the activity or protein expression of MMP-2, MMP-9, TIMP-1, TIMP-2, uPA, uPAR and PAI-1, and patient Dukes' stage, cellular differentiation, or lymphovascular invasion status was found.

## Conclusion

In summary, the present study indicates that the activities or protein levels of MMP-2, MMP-9, TIMP-1, TIMP-2, uPAR and PAI-1 do not differ between tumors located in the colon or rectum. However, uPA may be expressed differentially in colon and rectal cancer.

## Abbreviations

MMP = matrix metalloproteinase; TIMP = tissue inhibitor of metalloproteinase; uPA = urokinase-type plasminogen activator; uPAR = urokinase-type plasminogen activator receptor; PAI = plasminogen activator inhibitor

## Competing interests

The author(s) declare that they have no competing interests.

## Authors' contributions

T-DK participated in the design of the study, performed statistical analysis, generated experimental data and drafted the manuscript. K-SS performed computational analyses. GL and HC generated experimental data. H-DP, KL, B-DH were participated in the review and commentary of documents relative to the study and manuscript editing. W-HY had the initial idea for the study and participated in the design of the study and its coordination and helped to draft the manuscript. All authors read and approved the final manuscript.

## Pre-publication history

The pre-publication history for this paper can be accessed here:



## Figures and Tables

**Table 1:** Patient characteristics

	Mean age (range)	Sex^b ^(n)	Location (n)	Dukes' stage (n)	Differentiation^a ^(n)	V/L^c ^(n)
Colon	64.0 (48–81)	m (7)	Caecum (5)	B (4)	moder. diff (8)	+/+ (12)
			Sigmoid (5)	C (7)		
		f (5)	Transverse (2)	D (1)	poorly diff (4)	
Rectum	68.2 (53–76)	m (7)	(10)	A (2)	moder. diff (10)	+/+ (10)
				B (4)		
		f (3)		C (4)	poorly diff (0)	

^a ^moder. diff = moderate differentiation, poorly diff = poorly differentiation

^b ^m = male, f = female

^c ^V/L = vascular invasion/lymphatic invasion
